# Protective effect of quercetin against the metabolic dysfunction of glucose and lipids and its associated learning and memory impairments in NAFLD rats

**DOI:** 10.1186/s12944-021-01590-x

**Published:** 2021-11-17

**Authors:** Xin-Ran Gao, Zheng Chen, Ke Fang, Jing-Xian Xu, Jin-Fang Ge

**Affiliations:** 1 School of Pharmacy, Anhui Medical University, 81 Meishan Road, Hefei, 230032 People’s Republic of China; 2 The Key Laboratory of Anti-inflammatory and Immune Medicine, Ministry of Education, Anhui Medical University, Hefei, China; 3 Anhui Provincial Laboratory of Inflammatory and Immune Disease, Anhui Institute of Innovative Drugs, Hefei, China

**Keywords:** NAFLD, Quercetin, TREM1/2, Glucose and lipids metabolism, Central nervous system, Cognitive function

## Abstract

**Background:**

Quercetin (QUE) is a flavonol reported with anti-inflammatory and antioxidant activities, and previous results from the group of this study have demonstrated its neuroprotective effect against lipopolysaccharide-induced neuropsychiatric injuries. However, little is known about its potential effect on neuropsychiatric injuries induced or accompanied by metabolic dysfunction of glucose and lipids.

**Methods:**

A nonalcoholic fatty liver disease (NAFLD) rat model was induced via a high-fat diet (HFD), and glucolipid parameters and liver function were measured. Behavioral performance was observed via the open field test (OFT) and the Morris water maze (MWM). The plasma levels of triggering receptor expressed on myeloid cells-1 (TREM1) and TREM2 were measured via enzyme-linked immunosorbent assay (ELISA). The protein expression levels of Synapsin-1 (Syn-1), Synaptatogmin-1 (Syt-1), TREM1 and TREM2 in the hippocampus were detected using western blotting. Morphological changes in the liver and hippocampus were detected by HE and Oil red or silver staining.

**Results:**

Compared with the control rats, HFD-induced NAFLD model rats presented significant metabolic dysfunction, hepatocyte steatosis, and impaired learning and memory ability, as indicated by the increased plasma concentrations of total cholesterol (TC) and triglyceride (TG), the impaired glucose tolerance, the accumulated fat droplets and balloon-like changes in the liver, and the increased escaping latency but decreased duration in the target quadrant in the Morris water maze. All these changes were reversed in QUE-treated rats. Moreover, apart from improving the morphological injuries in the hippocampus, treatment with QUE could increase the decreased plasma concentration and hippocampal protein expression of TREM1 in NAFLD rats and increase the decreased expression of Syn-1 and Syt-1 in the hippocampus.

**Conclusions:**

These results suggested the therapeutic potential of QUE against NAFLD-associated impairment of learning and memory, and the mechanism might involve regulating the metabolic dysfunction of glucose and lipids and balancing the protein expression of synaptic plasticity markers and TREM1/2 in the hippocampus.

## Background

Nonalcoholic fatty liver disease (NAFLD) is a chronic liver injury characterized by excessive intrahepatic fat accumulation without increased alcohol consumption or other causes of liver steatosis. Due to alterations in lifestyle and the increasing incidence of obesity, the prevalence of NAFLD is increasing worldwide. In China, NAFLD has been reported to be the second most common liver disease following viral hepatitis [1]. Moreover, apart from metabolic dysfunction and liver injuries, which are the main clinical manifestation of NAFLD, an increasing incidence of neuropsychiatric diseases, including cognitive dysfunction [2] and depression [3], are reported in NAFLD patients, together with decreased brain volume and pathological changes [4]. The results of animal studies have also demonstrated oxidative stress and disturbances of neurotransmitter activities in the brains of NAFLD rats [5]. In line with these findings, HFD-induced NAFLD rats presented not only metabolic dysfunction, including obesity, dyslipidemia, glucose metabolic dysfunction, and liver dysfunction but also behavioral performance of impaired learning and memory [6], although the mechanism remains unclear. These results suggested a close association between NAFLD and neuropsychiatric impairments [7]. Thus, it is imperative to further investigate the pathogenesis of NAFLD and explore potential therapeutic drugs with high efficacy and few side effects.

Hyperlipemia, together with its resulting or accompanied insulin resistance, has been considered an important risk factor triggering neuropsychiatric injuries in metabolic diseases, including diabetes mellitus and NAFLD. Cholesterol is a component of neuronal cell membranes in the brain, and glucose is an important energy source for the brain. Dysfunction of glucose and lipid metabolism can affect the normal transmission of signal molecules between synapses and lead to learning and memory impairment and cognitive disorder [8]. It has been demonstrated in animal research that hyperinsulinemia, especially elevated serum cholesterol and triglyceride levels, are negative factors that are closely related to the cognitive performance of rats in the MWM task [9] or the novel object recognition (NOR) test [10]. Moreover, sitagliptin (STG), a common antidiabetic drug targeting glucagon-like peptide-1 (GLP-1) signaling via dipeptidyl peptidase 4, is reported not only to improve glucose and lipid metabolism disorders but also to have a protective effect on neuropsychiatric injuries in diabetes mellitus [11] patients or HFD mice [12]. Thus, the regulation of glucose and lipid metabolism might be a fundamental treatment for NAFLD and its associated neuropsychiatric injury.

Synaptic plasticity plays a vital role in maintaining normal brain structure and functions [13], and a decreased abundance of synaptic proteins has been identified to be involved in the pathophysiology of neuropsychiatric diseases, including mild cognitive impairment, Alzheimer’s disease (AD), and depression [14, 15]. Synaptotagmin-1 (Syt-1) and Synapsin-1 (Syn-1) are crucial synaptic-associated proteins for vesicle fusion, neurotransmitter release, and synaptic transmission [16, 17]. In the previous study, imbalanced expression of Syn-1 and Syt-1 was presented in the hippocampus and prefrontal cortex (PFC) in chronically stressed [18, 19] or subclinical hypothyroidism (SCH) suffering rats [20] and was significantly correlated with depression-like behaviors and impaired learning and memory. Consistently, decreased Syt-1 levels have also been found in the hippocampus of rats that experienced chronic stress or long-term corticosterone administration [21, 22]. However, little is known about their roles in NAFLD-associated neurobiological injuries.

The triggering receptors expressed on myeloid cells (TREMs) are members of the immunoglobulin family [23], with the main pathophysiological function in regulating inflammatory and immune responses. In recent years, much research has focused on the role of TREM1/2 in neuropsychiatric diseases, including AD, although the results have not always been consistent [24–26]. Mounting evidence has demonstrated that many pathological conditions can alter the balance in TREM expression, resulting in separate outcomes from distinct diseases [27]. Consistently, carried out by the same group of this study, the results of a previous study demonstrated behavior-related variations in the hippocampal abundance of TREM2 between rats aged 2 months and 6 months. Moreover, decreased expression of TREM1 but increased expression of TREM2 were also demonstrated in the hippocampus and PFC of rats challenged with lipopolysaccharide (LPS), accompanied by depression-like behaviors and impairment of learning and memory [28]. In addition, an imbalance of TREM1 and TREM2 has also been reported to be involved in the pathological process and therapeutic outcome of nonalcoholic steatohepatitis (NASH) [29]. These results suggest that the abundance or function of TREM1 and TREM2 might be one of the potential targets for improving NAFLD, not only the metabolic dysfunction of glucose and lipid and liver injuries but also neuropsychiatric impairments.

Querctin (QUE) is a flavonol derived from diverse plants. It has been reported that QUE could ameliorate glucose tolerance and reduce serum concentrations of insulin, low-density lipoprotein cholesterol (LDL), and triacylglycerol in HFD rats [30]. Targeting its neuroprotective effect, the results of a previous study showed that QUE could bind to mono- or fibril amyloid beta and alleviate LPS-induced depressive behaviors and impaired learning and memory in rats [28], which was in line with the findings of other studies [31]. These results indicated that QUE might be a good strategy for therapeutic intervention against NAFLD, targeting both metabolic dysfunction and behavioral impairments.

The purpose of the present study was to investigate the potential effect of QUE against metabolic and neuropsychiatric injuries in NAFLD and explore the possible mechanism. An NAFLD rat model was established via HFD, and plasma glucose and lipid concentrations and liver function were measured. Behavioral performance was examined using the open field test (OFT) and the Morris water maze (MWM). Morphological changes in the liver and hippocampus were detected through HE, Oil red, or silver staining. Moreover, the protein expression levels of syt-1, syn-1 and TREM1/2 in the hippocampus were measured using western blotting.

## Materials and methods

### Drugs

QUE was purchased from Sigma Chemical Co. (Louis, MO, USA). Sitagliptin phosphate was produced by Merck Sharp Dohme Ltd. (New Jersey, USA). Donepezil hydrochloride was produced by Jiangsu Haosen Pharmaceutical Co., Ltd. (Jiangsu, China). All the drugs were dissolved in 0.5% sodium carboxymethyl cellulose aqueous solution to make a mixed suspension.

### Animals and groups

Sixty-four Sprague–Dawley (SD) male rats (aged 2 months) were purchased from the experimental animal center of Anhui Medical University (Anhui, China). They were fed under ambient temperature conditions of 21–22 °C and 50–60% relative humidity with a commutative 12 h light/dark schedule. All animal care practices and experimental procedures were approved by the Animal Care and Use Committee of Anhui Medical University in compliance with the National Institutes of Health Guide for the Care and Use of Laboratory Animals (NIH publication No. 85–23, revised 1985).

**Fig. 1:**
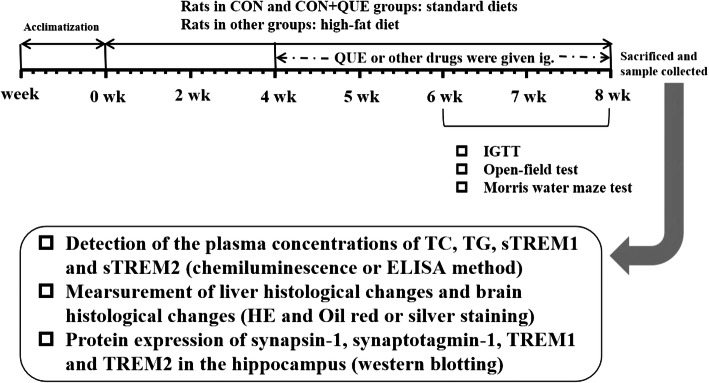
Outline of the experimental design for nonalcoholic fatty liver disease (NAFLD) and behavioral tests

After 1 week of acclimatization, the rats were randomly divided into eight groups, including the control group (CON), CON+QUE (40 mg/kg) group, NAFLD group, NAFLD+QUE (80, 40, or 20 mg/kg) group, NAFLD+STG (sitagliptin, 10 mg/kg) group, and NAFLD+DNP (donepezil, 1 mg/kg) group, with 8 rats in each of groups and 4 rats per cage, access to food and water at liberty. The diets fed to the rats were as described in a previous study [6]. Briefly, the rats in the CON and CON+QUE groups were administered a standard diet (3601 kcal/kg; Trophic Animal Feed High-Tech Co., Ltd., Nantong, China), and rats in other groups were given a high-fat diet (5000 kcal/kg; Trophic Animal Feed High-Tech Co., Ltd., Nantong, China). Food consumption was measured every day and change in body weight was measured every week. The energy consumption of rats in the CON and CON+QUE groups was calculated by the food consumption × 3.601 kcal, and rats in other groups were calculated by the food consumption × 5.000 kcal. After 4 weeks, QUE, STG and DNP were administered intragastrically for 4 weeks. Rats in the control and untreated NAFLD groups received the corresponding volume of 0.5% sodium carboxymethyl cellulose via the same route. The outline of the experimental design is shown in Fig. 1.

### Behavioral tests

All behavioral tests were performed according to a previous study [6], with test time matched between groups. Before each test, all the animals were given an adaptive time of 30 min to the testing environment, and the observers were blinded to the group arrangement. A digital camera interfaced with a computer equipped with ANY-maze video imaging software (Stoelting Co, Wood Dale, USA) was used to monitor and record the behavioral performance.

### Open-field test (OFT)

The OFT experimental apparatus is composed of a 100 cm square field, and the floor is divided into 16 small squares with equal grids. The device is surrounded by a 30 cm high wall, and the entire device is black. During the test, each rat was placed in a corner of the apparatus facing the wall and allowed to move free for 5 min. The frequencies of rearing, grooming and defecation were recorded for statistical analysis.

### Morris water maze (MWM) test

The circular black pool for the Morris water maze is 180 cm in diameter and 50 cm in height, and there are several large visual cues constantly surrounding the tank at a height of 120–150 cm. Before the test, the pool was filled with water to 40 cm high, and the water was dyed black with an edible pigment. A circular escape platform was hidden 1 cm under the surface of the water. The circular water surface was divided into four equal quadrants, and the quadrant where the escape platform was located was defined as the target quadrant.

The task consisted of a 3-day acquisition phase with 4 massed trials administered each day and a 1-day probe phase on the 4th day. During the acquisition phase, the platform was invisible and constant, and the 4 start locations were distributed north, south, east and west, located at equal distances from each other on the pool rim. Each rat was placed in the water facing the wall atone random start location and allowed to find the submerged platform within 60 s and rest there for 30 s; then, the next trial began. If the rat failed to find the hidden platform within 60 s, it was guided to the platform and allowed to remain there for 30 s. The procedure was repeated for all four start locations, and the escape latency (latency to find the platform) was recorded.

On the 4th day, the platform was removed, and the probe test was performed to assess memory retention. Each rat was put into the pool from the diagonally opposite side of the previous platform and allowed to explore the environment for 60 s ad libitum. The duration spent in the target quadrant in the probe test were analyzed.

### Intraperitoneal glucose tolerance test (IGTT)

After fasting and water prohibition for 12 h, each rat was given an intraperitoneal injection of glucose at a dose of 2.0 g/kg. Tail tip blood was collected before injection and 15, 30, 60 and 120 min after injection, and a Roche glycemic meter was used to measure the blood glucose concentration.

### Measurement of the plasma concentrations of TC, TG, sTREM1, and sTREM2

Twenty-four hours after the MWM test, the rats were fasted overnight and deeply anesthetized with chloral hydrate. Blood samples were drawn from the abdominal aorta and centrifuged at 3000 rpm for 30 min at 4 °C. The plasma concentrations of total cholesterol (TC) and triglyceride (TG) were detected by Adicon Medical Laboratory Center (Adicon clinical laboratories, LTD, Hefei, China) using chemiluminescence kits (Nanjing Jiancheng Bioengineering Institute, Nanjing, China). The plasma levels of sTREM1 and sTREM2 were measured via commercially available enzyme-linked immunosorbent assay (ELISA) kits (sTREM1 and sTREM2: Huamei Biotech. Co., LTD, Wuhan, China) according to the manufacturers’ instructions.

### Measurement of the liver index and histological changes

After the blood was collected, the liver was dissected and weighed, and the liver index was calculated by the liver weight/100 g body weight. Then, four rats from each group were randomly selected, and the same parts of the livers were collected and fixed in 4% neutral-buffered formalin. After dehydration with graded ethanol, the livers were embedded in paraffin and cut into 4 μM thick sections. The sections were stained with hematoxylin and eosin (HE staining) or 0.5% oil red O (oil red O staining) at room temperature. Photomicrographs were captured with an IX51 microscope (Olympus Corporation, Tokyo, Japan).

### Measurement of the histological changes in the hippocampus

Four rats in each group were randomly selected, and the whole brain was collected, fixed with 4% neutral-buffered formalin, embedded in paraffin, sectioned at a thickness of 4 μM, and stained with hematoxylin and eosin (HE staining) and silver nitrate (silver staining) according to the regular procedure. Briefly, silver staining was conducted as follows: the tissue was circled with an immunohistochemical pen, then 5% silver nitrate was dropped onto the tissue and reacted with wet box for 20 min; then prepared silver ammonia solution drops on the tissue and soaked in dilute ammonia water; the developer was prepared and reacted with the tissue for approximately 5 min until the nerve fibers appeared black. Photomicrographs were captured with an IX51 microscope (Olympus Corporation, Tokyo, Japan).

### Western blotting

The hippocampus from the other four rats in each group were rapidly dissected, frozen in liquid nitrogen, and stored at − 80 °C. The tissues were homogenized in radioimmunoprecipitation assay (RIPA) buffer (Beyotime Biotechnology, Shanghai, China). Prior to homogenization, protease inhibitor cocktail (Sigma, St. Louis, MO, USA) and phosphatase inhibitor PhosSTOP (Roche, Indianapolis, USA) was added. Protein was quantified via a Lowry Protein Assay Kit (Meiji Biotech. Co., LTD, Shanghai, China). A total of 50 μg protein from each sample was loaded, separated via 12% SDS–PAGE gels, and then transferred to PVDF membranes (Millipore, IPVH00010). After blocking with 5% skim milk for 2 h, the membrane was incubated with the corresponding antibodies targeting synapsin-1 (1:1000, Immunoway, Newark, DE, USA), synaptotagmin-1 (1:1000, Immunoway, Newark, DE, USA), TREM1 (1:1000, Abcam, Cambridge, UK), TREM2 (1:1000, Abcam, Cambridge, UK) or β-actin (1:1000, Zhongshan Biotechnology, Inc., Beijing, China) at 4 °C overnight. Subsequently, the membranes were processed with appropriate HRP-conjugated secondary antibodies (1:5000, Zhongshan Biotechnology, Inc., Beijing, China) with regard to the proteins of interest at room temperature for 1 h. The protein levels were analyzed using ImageJ (Wayne Rasband, National Institutes of Health, USA) and normalized relative to that of the internal control β-actin.

### Statistical analysis

All statistical analyses were performed using SPSS (Statistical Package for the Social Sciences, version 17.0.1, SPSS Inc., Chicago, IL, USA). Data are expressed as the mean ± SEM., and *P* < 0.05 was considered statistically significant. Statistical analysis between groups was conducted by one-way analysis of variance (ANOVA) followed by the LSD post hoc test.

## Results

### QUE alleviated hyperglycemia, hyperlipemia, hepatomegaly, and hepatocyte steatosis in NAFLD rats

**Fig. 2:**
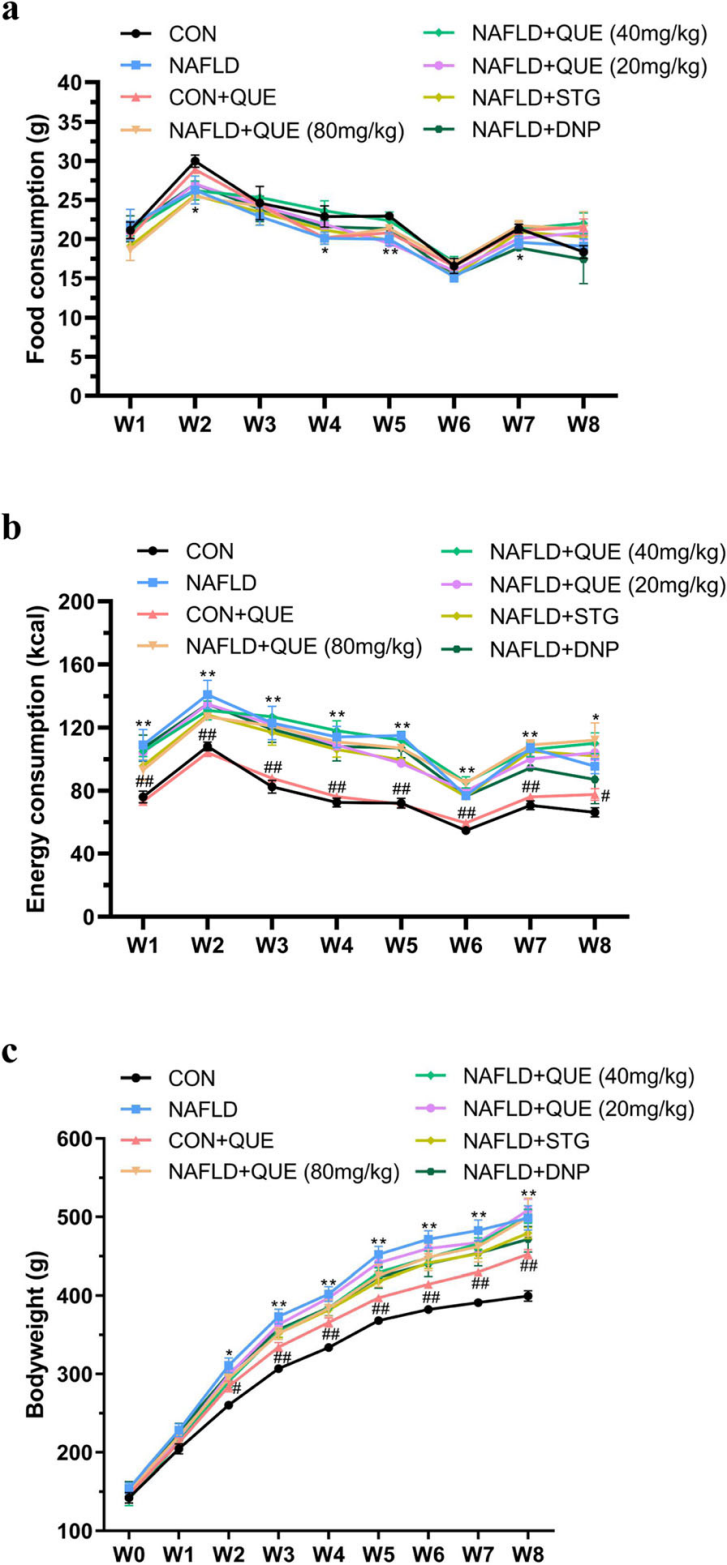
Effect of QUE on the food consumption, energy consumption and body weight of NAFLD rats. The data are presented as the mean ± SEM, with 8 rats in each of the groups. The NAFLD model rats showed less food consumption (**a**) but greater energy consumption (**b**) and greater body weight (**c**) than the control or CON+QUE rats. However, treatment with QUE (80, 40, or 20 mg/kg) had no significant effect on these changes. ^*^P < 0.05 and ^**^P < 0.01 compared with the control group. ^#^*P* < 0.05 and ^##^*P* < 0.01 compared with the NAFLD model group

Figure 2 shows the changes in food consumption (Fig. 2a), energy consumption (Fig. 2b), and body weight (Fig. 2c) in all the groups. Although the quantity of food consumption in the NAFLD model group was less than that of the controls, the energy consumption and body weight gain were both significantly increased. However, there was no significant difference between the NAFLD model group and the QUE-, STG-, or DNP-treated groups with regard to food consumption, energy consumption, and body weight.

**Fig. 3:**
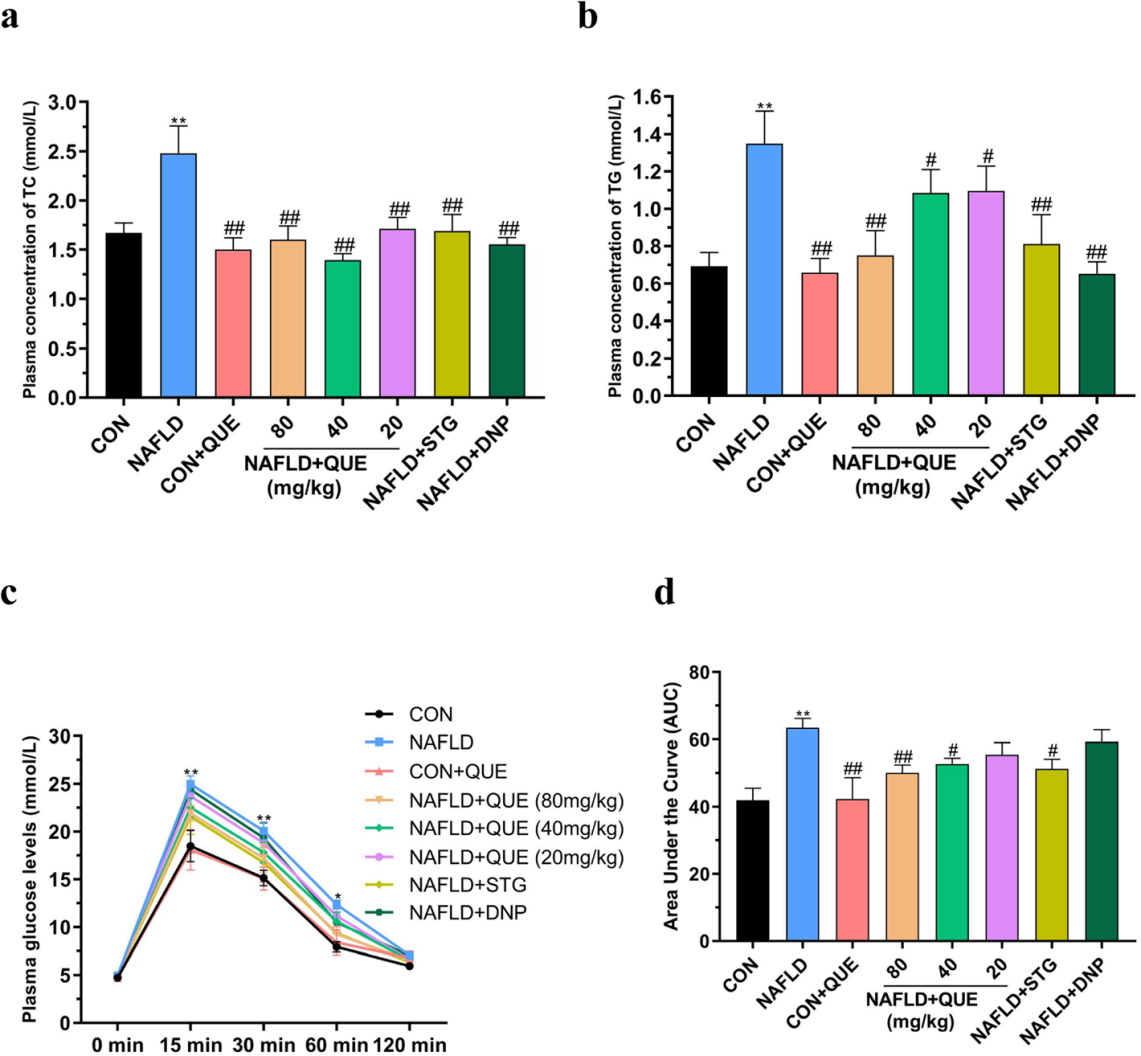
Effect of QUE on the plasma concentrations of TC and TG and IGTT in NAFLD rats. The data are presented as the mean ± SEM, with 8 rats in each of the groups. Compared with the control and CON+QUE rats, the NAFLD group showed increased plasma TC (**a**) and TG (**b**) levels, and treatment with QUE decreased the plasma concentrations of TC and TG. Treatment with QUE also improved the impaired glucose tolerance of NAFLD rats (**c, d**). ^*^P < 0.05 and ^**^P < 0.01 compared with the control group. ^#^*P* < 0.05 and ^##^*P* < 0.01 compared with the NAFLD model group

Compared with those in the control group, the plasma concentrations of TC (Fig. 3a) and TG (Fig. 3b) were significantly increased in the NAFLD group. There was no significant difference between the CON and CON+QUE groups. However, the plasma concentrations of TC (Fig. 3a) and TG (Fig. 3b) were remarkably decreased in the QUE-, STG-, or DNP-treated group compared with the NAFLD model group. As shown in Fig. 3c and d, the blood glucose levels of 15-, 30- and 60-min after intraperitoneal injection of glucose in the IGTT were remarkably increased in the NAFLD rats as compared with those of the control and CON+QUE groups. Treatment with QUE also improved the impaired glucose tolerance of NAFLD rats.

**Fig. 4:**
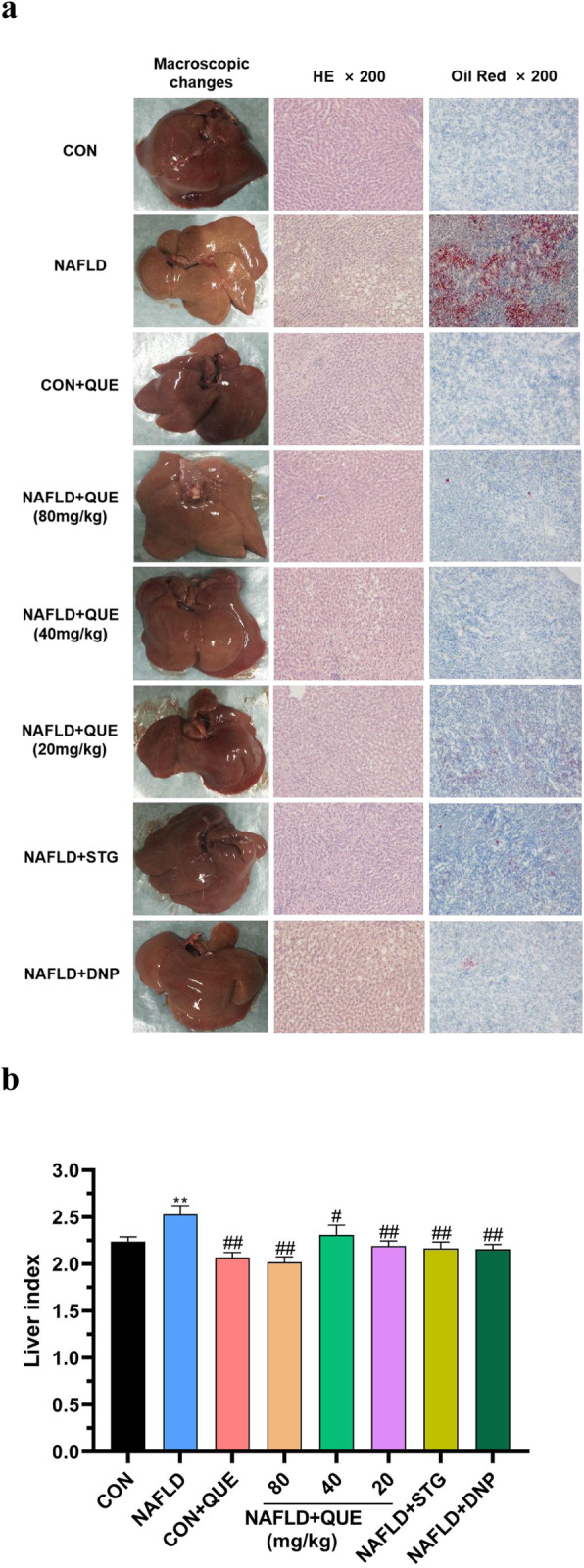
Effect of QUE on macroscopic and histological liver changes in NAFLD rats. The data are presented as the mean ± SEM, with 8 rats in each of the groups. The livers of the NAFLD rats appeared yellow or pale, greasy, and brittle. Inflammatory cells and numerous lipid droplets were detected in the livers of NAFLD rats via HE staining. The macroscopic and histological liver changes in the QUE-treated group were significantly decreased. Compared with the control and CON+QUE rats, the NAFLD group also showed increased liver index (**b**), and treatment with QUE reversed the increase in the variables. ^**^P < 0.01 compared with the control group. ^#^*P* < 0.05 and ^##^*P* < 0.01 compared with the NAFLD model group

Figure 4a shows the macroscopic and microscopic histological changes in the livers of all the groups. Compared with that of the control group, the livers of the NAFLD rats appeared yellow or pale, greasy, and brittle. The results of HE and oil red staining revealed aggregation of inflammatory cells and numerous lipid droplets in the livers of NAFLD rats, which was lightened in the NAFLD+QUE group. As shown in Fig. 4b, the liver index were also increased in the NAFLD group compared with the control group. Treatment with QUE reversed the increase of liver index.

### QUE improved the impaired learning and memory ability of NAFLD rats

**Fig. 5:**
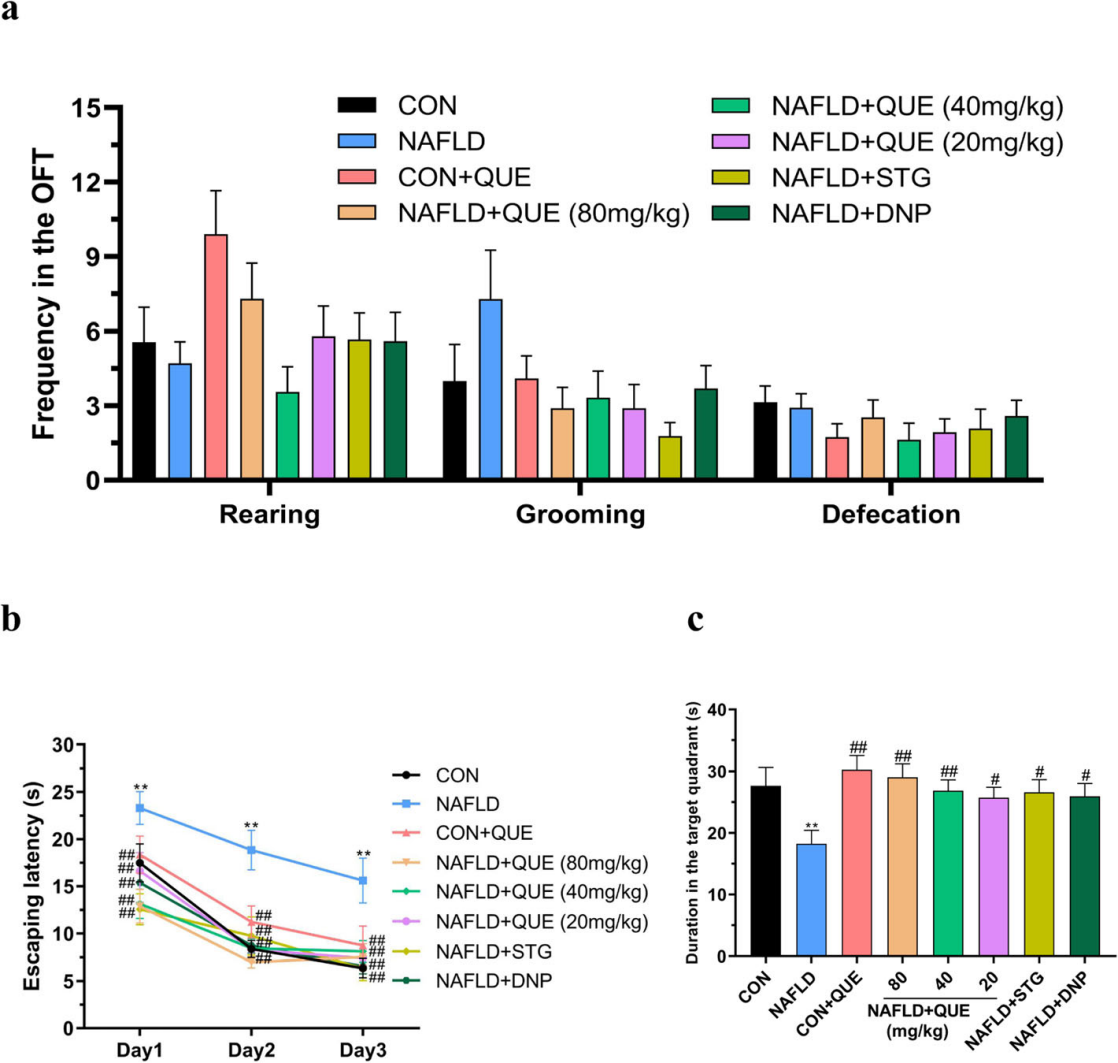
Effect of QUE on the behavioral performance of NAFLD rats in the OFT and MWM. The data are presented as the mean ± SEM, with 8 rats in each of the groups. In the OFT, there was no significant difference among groups with respect to the frequency of rearing, grooming or defecation (**a**). In the MWM, the escape latency of the NAFLD rats was longer than that of the control or CON+QUE rats on all 3 days of the acquisition phase (**b**). In the probe trial, NAFLD rats spent less time than control or CON+QUE rats in the target quadrant (**c**). These abnormalities could be reversed by treatment with QUE. ^**^P < 0.01 compared with the control group. ^#^*P* < 0.05 and ^##^*P* < 0.01 compared with the NAFLD model group

Figure 5 shows the performance of rats in the behavioral tasks. In the OFT, there was no significant difference among groups in the rearing frequency, grooming frequency or defecation frequency (Fig. 5a).

In the MWM task, the escape latency of all the rats in this study declined gradually in the acquisition phase (Fig. 5b), indicating that all rats could fulfill the learning task. However, NAFLD rats spent a longer time finding the submerged platform than did the control or CON+QUE rats on all 3 days. In the probe test (Fig. 5c), the duration in the target quadrant of the NAFLD rats was remarkably less than that of the control and CON+QUE rats. However, these impairments were reversed by treatment with QUE.

### QUE alleviated morphological injury in the hippocampus of NAFLD rats

**Fig. 6:**
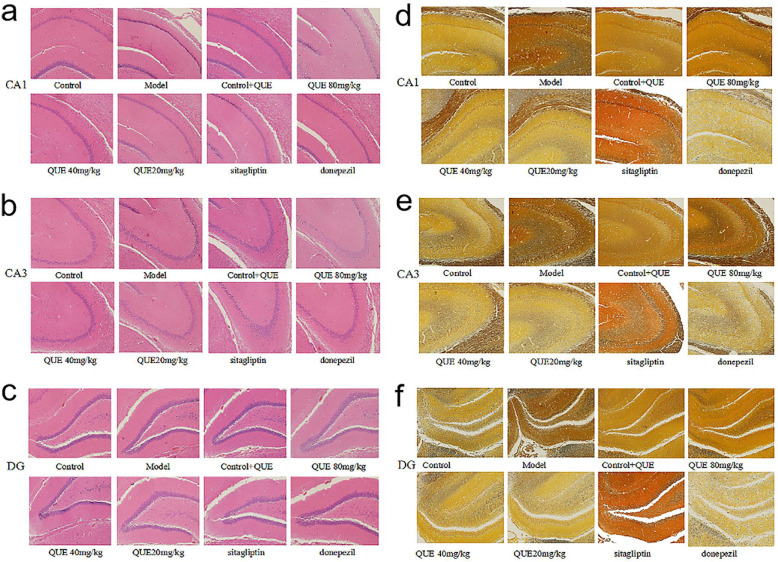
Effect of QUE on morphological injury in the hippocampus of NAFLD rats. (**a ~ c**) show the HE staining results, and (**d ~ f**) show the silver staining results. The neuronal cells of the NAFLD rats were irregular and loosely arranged, and the cell morphology was atrophic and hyperchromatic, with unclear boundaries of nuclei and cytoplasm. The morphological structure of the hippocampus in the NAFLD rats treated with QUE was similar to that of the normal rats

Figure 6 shows typical HE and silver staining graphs of the CA1, CA3, and DG regions of the hippocampus in each group. The results of HE staining showed that the neurons in the hippocampus of the control and CON+QUE groups presented with regular shapes and compact arrangements, round and large nuclei, clearly visible nucleoli, and no deep staining of neurons in the sections. However, in the model group, the neuronal cells in each district were irregular and loosely arranged, and the cell morphology was atrophic and hyperchromatic, with unclear boundaries of nuclei and cytoplasm (Fig.6a to 6c). Silver staining showed that compared with the control and control+QUE groups, the nerve cells and nerve fibers of the model group were dyed black, which suggested neuronal damage in the model group (Fig. 6d to 6f). The morphological structure of the hippocampus in the NAFLD rats treated with QUE was similar to that of the normal rats.

### QUE balanced the protein expression of Syn-1 and Syt-1 in the hippocampus of NAFLD rats

**Fig. 7:**
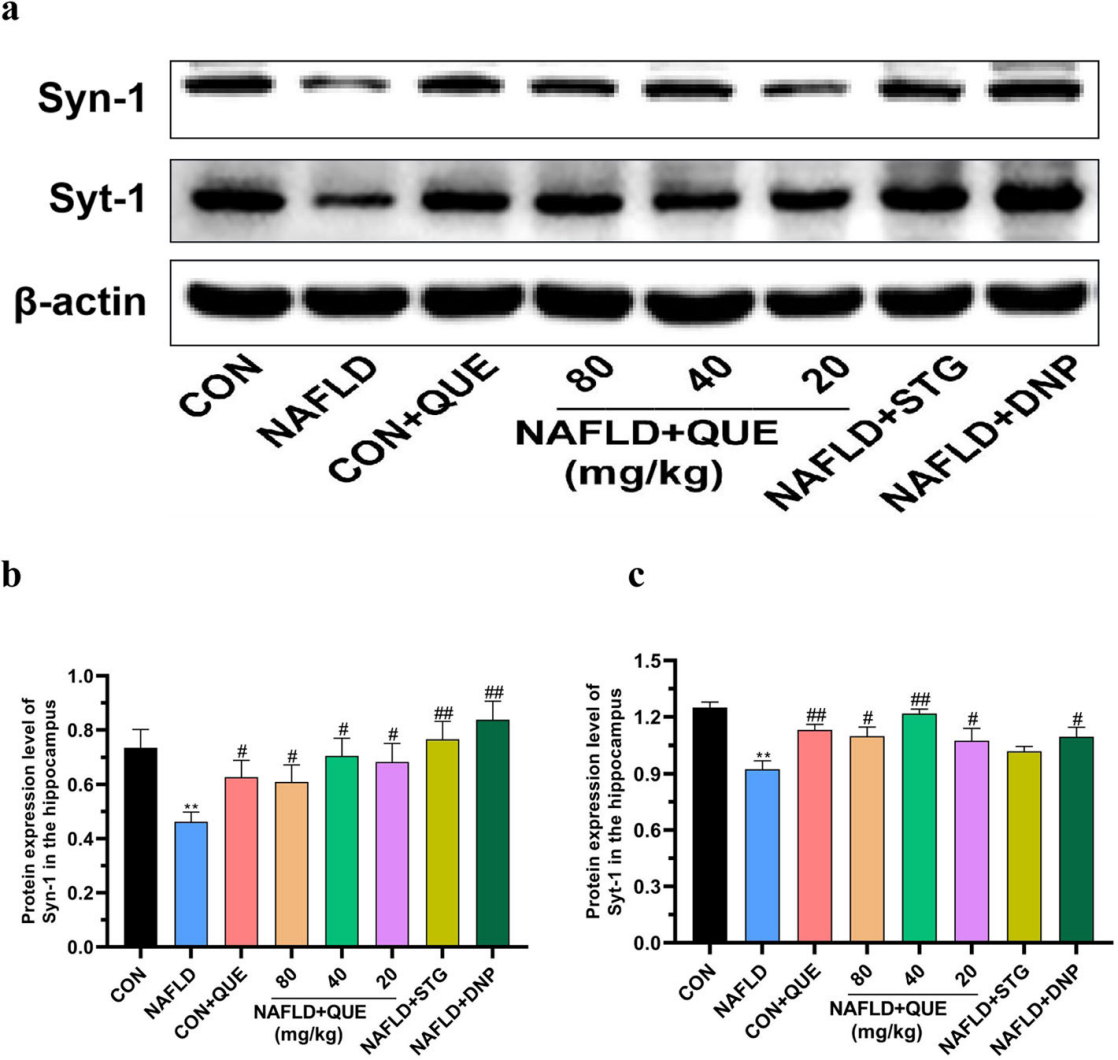
Effect of QUE on the protein expression of Syn-1 and Syt-1 in the hippocampus of NAFLD rats. (**a**) shows a typical graph, and (**b ~ c**) show a statistical analysis of the western blotting results. The data in (**b ~ c**) are presented as the mean ± SEM, with *n* = 3 for each group. The protein expression of Syn-1 and Syt-1 was decreased in the hippocampus of NAFLD. Treatment with QUE restored the imbalanced expression of Syn-1 and Syt-1 to normal in the hippocampus. ^**^P < 0.01 compared with the control group. ^#^*P* < 0.05 and ^##^*P* < 0.01 compared with the NAFLD model group

As shown in Fig. 7, compared with that in the control group, the protein expression of Syn-1 (Fig. 7b) and Syt-1 (Fig. 7c) was decreased in the hippocampus of NAFLD rats. Treatment with QUE imbalanced the protein expression of Syn-1 and Syt-1 in the hippocampus.

### QUE balanced the abundance of TREM1 in the blood and hippocampus of NAFLD rats, but TREM2 was balanced only in the hippocampus

**Fig. 8:**
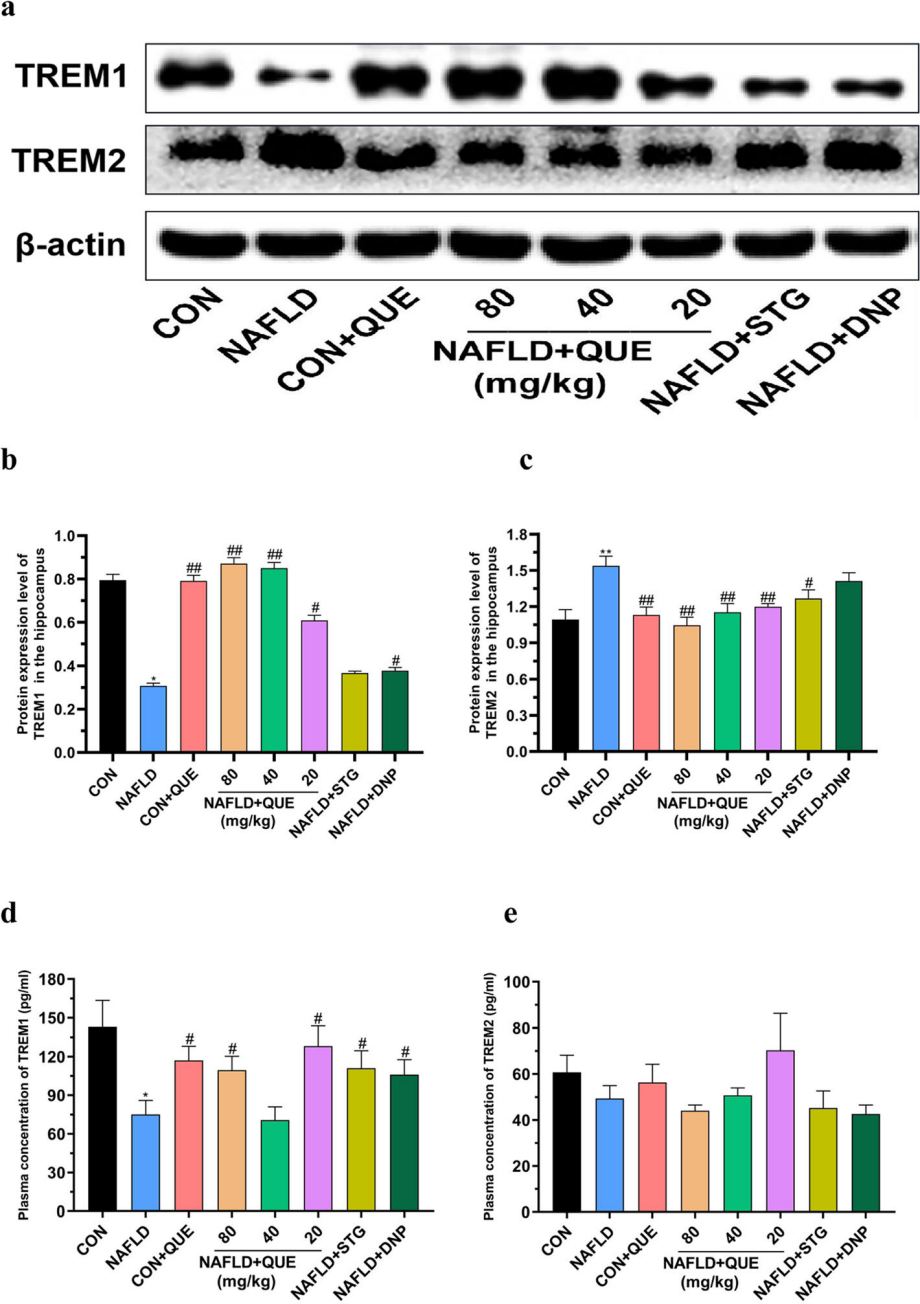
Effect of QUE on the expression of TREM1 and TREM2 in the plasma and hippocampus of NAFLD rats. (**a**) shows a typical graph, and (**b ~ c**) show a statistical analysis of the western blotting results. (**d ~ e**) shows the plasma concentrations of TREM1 and TREM2 in NAFLD rats. The data in (**b ~ c**) are presented as the mean ± SEM, with *n* = 3 for each group. The data in (**d ~ e**) are presented as the mean ± SEM, with *n* = 8 for each group. The protein expression of TREM1 was decreased, whereas the protein expression of TREM2 was increased in the hippocampus of NAFLD rats. Treatment with QUE reversed the imbalanced expression of TREM1 and TREM2. Compared with the control and CON+QUE rats, the NAFLD group showed decreased plasma TREM1 (**d**) levels, while the plasma TREM2 (**e**) level was not changed. Treatment with QUE increased the plasma concentrations of TREM1 but had no effect on the plasma TREM2 level. ^*^P < 0.05 and ^**^P < 0.01 compared with the control group. ^#^*P* < 0.05 and ^##^*P* < 0.01 compared with the NAFLD model group

Figure 8 shows the abundance of TREM1 and TREM2 in the plasma and hippocampus of rats in all the groups. Compared with that in the control group, the protein expression of TREM1 (Fig. 8b) was decreased but that of TREM2 (Fig. 8c) was increased in the hippocampus, and treatment with QUE reversed the imbalanced expression of TREM1 and TREM2. Consistently, the plasma concentrations of TREM1 (Fig. 8d) in the model group were remarkably lower than those in the control or CON+QUE groups, and treatment with QUE reversed the changes in TREM1. However, there was no significant difference among the groups in the plasma concentration of TREM2 (Fig. 8e).

## Discussion

**Fig. 9:**
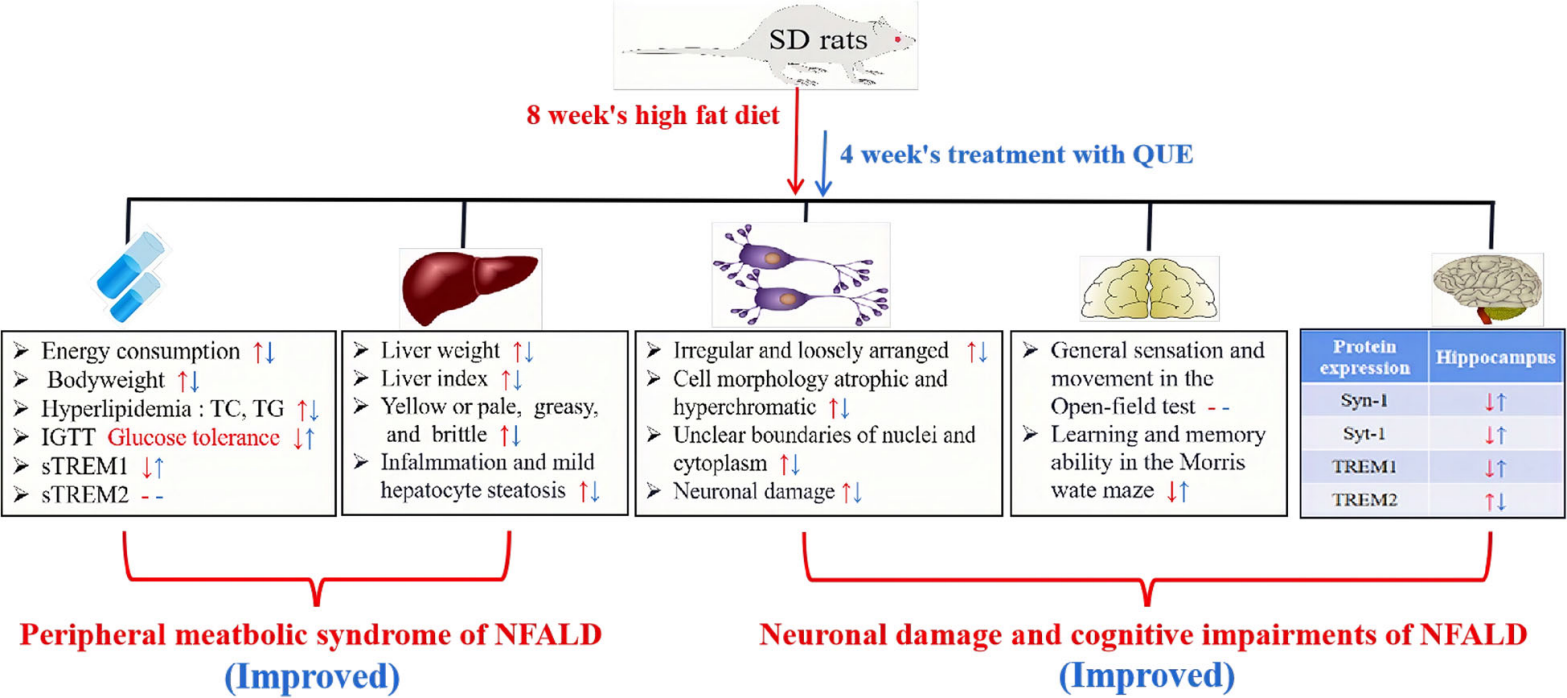
Summary of the therapeutic effect of QUE on peripheral metabolic syndrome and behavioral and cognitive impairments associated with NAFLD in a rat model. The red description and arrows indicate the dysfunction induced by 8 weeks on a high-fat diet, and the blue ones show the effects of 4 consecutive weeks of treatment with QUE.

The results of the present study showed that administration of QUE could exert a therapeutic effect against NAFLD in rats, not only including glucose and lipid metabolism disorders and liver injuries but also impaired learning and memory. The mechanism might be associated with its ability to improve lipid metabolism, regulate synaptic plasticity, and balance the abundance of TREM1 and TREM2. Figure 9 shows the summary of the therapeutic effect of QUE against the metabolic dysfunction and cognitive impairments of NAFLD model rats.

Hyperglycemia, hyperlipidemia, and liver dysfunction are the main clinical manifestations of NAFLD. Consistent with previous findings [6], the plasma concentrations of TG and TC in rats fed a high-fat diet were remarkably increased in this study, together with obesity, impaired glucose tolerance, hepatomegaly, hepatocyte steatosis, and liver dysfunction. These results again indicated the causal link between diet and metabolic disorders and that a high-fat diet could be used in establishing NAFLD animal models.

Sitagliptin (STG) is a representative DPP-4 inhibitor and is widely used in treating type 2 diabetes mellitus (T2DM) [32]. Considering the important role of glucose metabolism in maintaining homeostasis and body function, the role of STG has been explored gradually. It has been reported that STG could not only improve glucose and lipid metabolic disorders [33, 34] but also alleviate cognitive impairments [35] in NAFLD and T2DM patients. Hence, STG was selected as a positive control drug in the present study dosing according to the dose translation formula based on body surface area, and the results showed that STG (10 mg/kg) could significantly reverse the metabolic dysfunction and liver injury of NAFLD rats. Similar to the effect of STG, QUE (80, 40, 20 mg/kg) also restored the increased plasma TG and TC concentrations of NAFLD rats to normal, accompanied by the improvement of glucose tolerance and morphological liver injuries. These results indicated the potential therapeutic effect of QUE against the metabolic dysfunction of NAFLD rats. Although it is difficult to explain the mechanism thoroughly, it has been demonstrated that QUE could improve very low-density lipoprotein (VLDL) assembly and lipophagy in HFD-induced NAFLD rats via the inositol-requiring transmembrane kinase/endoribonuclease 1a/spliced X-box binding protein 1 (1IRE1a/XBP1s) pathway [36]. Moreover, QUE could also improve lipid metabolism by regulating the protein expression of sterol regulatory element-binding proteins-1 (SREBP1) [37], which is an important transcription factor involved in the lipid-associated impairments induced by fatty acids, triacylglycerols, cholesterol, and oleic acid [38–40]. These results suggested that QUE might be an active ingredient with multiple therapeutic targets.

Increasing evidence suggests a close correlation between NAFLD and neuropsychiatric diseases [3, 4, 41]. It has been reported that NAFLD can induce disturbances in neurotransmitter activities, accompanied by extensive oxidative stress and metabolic disorders in rats [5]. A clinical study conducted by Asuman et al. [2] showed that the MoCA scores of the participants with NAFLD were significantly lower than those of the healthy controls. In the present study, although there was no significant difference among groups of behavioral performance in the OFT, NAFLD rats showed an impairment of learning and memory ability in the MWM task, as indicated by the increased escape latency in the acquisition phase and the reduced duration in the target quadrant in the probe trial. As a clinically widely used regent with the efficacy of improving cognitive dysfunction in AD patients, donepezil (DNP) was also used as a positive control drug in this study, and the results unsurprisingly demonstrated that the impaired learning and memory of NAFLD rats was alleviated by treatment with DNP. In line with the effect of DNP, the NAFLD rats treated with QUE also spent less time finding the platform and more time in the target quadrant in the MWM task. These results suggested that QUE could improve the impairment of learning and memory ability in NAFLD rats.

Synaptic plasticity represents one of the most fundamental and important functions of the brain. As the main proteins involved in the regulation of synaptic plasticity, Syt-1 and Syn-1 are crucial for cognitive function [42–44]. Consistently, imbalanced expression of Syt-1 and Syn-1 was demonstrated in stressed or SCH rats in previous studies [20]. In the present study, decreased protein expression levels of Syt-1 and Syn-1 were present in the hippocampus of NAFLD rats, and treatment with QUE increased the levels of these two synaptic-related proteins. These results suggested that the change in synaptic plasticity might be one of the risk factors participating in the dysfunction of learning and memory in NAFLD rats and might be a possible therapeutic target for QUE.

The triggering receptor expressed on myeloid cells (TREMs) family consists of a group of cell surface innate immune receptors of the immunoglobulin superfamily and plays an important role in maintaining homeostasis and the normal immune response. Dou et al. demonstrated that the imbalance of TREM1 and TREM2 in the liver was involved in the pathological process of NASH, with a close association between the severity of fatty liver and the rise of TREM1 and reduction of TREM2 [29]. Targeting at the role of TREM1 and TREM2 in brain structure and brain diseases, many investigations have been conducted, and the results showed that the protein expression of TREM1 and TREM2 in the hippocampus and PFC could be altered by age or inflammatory stimulants [28]. Upregulating the expression of TREM2 could inhibit the apoptosis of hippocampal neurons [45], ameliorate neuropathology, and rescue spatial cognitive impairment [46]. Loss of TREM2 function could increase amyloid seeding [47] and result in aggravated spatial learning dysfunction in P301S transgenic mice [48]. In the present study, although there was no significant change in the plasma concentration of sTREM2, the NAFLD rats presented not only a declining abundance of TREM1 in the plasma and hippocampus but also an increased expression of TREM2 in the hippocampus. However, the chaos of TREM1/2 in the hippocampus could be reversed by treatment with QUE. These results show that imbalanced abundance of TREM1 and TREM2 may be involved in the pathological process of NAFLD, and QUE could improve the learning and memory impairment of NAFLD rats by reversing the abnormal expression of TREM1 and TREM2 in the hippocampus.

### Comparisons with other studies and what does the current work add to the existing knowledge

Several preclinical studies have suggested that HFD-induced NAFLD rats could initiate dysfunction of glucose and lipid metabolism [49], and QUE has a certain protective effect on neuropsychiatric injury [50, 51]. However, little was focusing on the protective effect of QUE against the neuropsychiatric injury induced by glucose and lipid metabolism disorders. The results of the current study revealed that QUE could improve learning and memory impairments in NAFLD rats, and the potential mechanism may be related to regulating hippocampal synaptic plasticity.

### Study strength and limitations

In the present study, there were several strengths. First, although the idea of the association between metabolic disease and neuropsychiatric injuries has been accepted, few studies have focused specifically on the neuropsychiatric injury induced by metabolic dysfunction of glucose and lipids in NAFLD. The findings of this study could help improve our understanding of the pathogenesis of NAFLD, especially its associated impairments in learning and memory. Second, the multiple effects of QUE against NAFLD were investigated via both biological markers and neurobiological methods, which could shed new light on the exploration of QUE against more therapeutic indications.

There were also several limitations in the present study. First, learning and memory ability was detected only through the MWM task. More behavioral tasks, such as the T maze or new object recognition (NOR), might be conducted in the same cohort of rats under the condition that confounding factors, including time, place, and order, should be taken into account and controlled. Second, the dose-effect relationship of QUE on the improvement of NAFLD rat indicators was not clear, and the mechanism leading to this phenomenon was not fully understood. One possible interpretation might be that the dose gradient was not large enough to distinguish, but the exact plasma concentration of QUE in each group was not detected in this study. Given the results that QUE exerts a protective effect without remarkably dose-effect in vitro in other studies [52], and the report that QUE was safely tolerated up to 2000 mg/day in human study [53], detailed investigation should be focused more on the dose selection in future research. Finally, although the therapeutic effect of QUE against peripheral metabolic dysfunction of glucose and lipid and neuropsychiatric injuries were both investigated in this study, detailed central metabolic changes and specific neuronal activity were not elaborated, which should be explored in our future study.

## Conclusions

The results of this study demonstrated that QUE could exert a therapeutic effect on a NAFLD rat model, alleviating abnormal glucose and lipid metabolism, relieving liver dysfunction, and improving behavioral impairments. The mechanism underlying the protective effect of QUE might be partly associated with its ability to improve lipid metabolism, regulate synaptic plasticity, and balance the abundance of TREM1 and TREM2.

QUE is a naturally-occurring flavonol with versatile pharmacological activities. With the deepening investigation, QUE are found to be suitable for increasing indications. Apart from its beneficial effect in preventing the progress of chronic obstructive pulmonary disease (NCT01708278), many clinical trials have been carried out focusing on its clinical benefits on prophylaxis and management of COVID-19 (NCT04377789, NCT05037240, and NCT04861298). Targeting its effect on metabolic disorders, a phase II clinical trials for the treatment of T2DM (NCT00065676) has been completed, and many benefit outcomes have also been reported [54]. Taken together with it properties of consumption as component of human diet and the therapeutic effect against metabolic dysfunction and cognitive impairments of NAFLD rats in this study, it is rational to infer that QUE, as well as its derivatives, will be developed as effective and safety drugs for treatment of metabolic disease and neuropsychiatric diseases from a clinical perspective.

## Data Availability

All data related to this study are available from the corresponding author (Professor JF Ge) upon request.

## Abbreviations

QUE: quercetin
NAFLD: nonalcoholic fatty liver disease
HFD: high-fat diet
OFT: open field test
MWM: Morris water maze
TREM1: triggering receptor expressed on myeloid cells-1
TREM2: triggering receptor expressed on myeloid cells-2
TC: total cholesterol
TG: triglyceride
Syn-1: Synapsin-1
Syt-1: Synaptatogmin-1
STG: sitagliptin
AD: Alzheimer’s disease
PFC: prefrontal cortex
NASH: nonalcoholic steatohepatitis
DNP: donepezil
SREBP1: sterol regulatory element-binding proteins-1
VLDL: very low-density lipoprotein
